# Robot-Supported Collaborative Learning (RSCL): Social Robots as Teaching Assistants for Higher Education Small Group Facilitation

**DOI:** 10.3389/frobt.2019.00148

**Published:** 2020-01-10

**Authors:** Rinat B. Rosenberg-Kima, Yaacov Koren, Goren Gordon

**Affiliations:** ^1^Faculty of Education in Science and Technology, Technion—Israel Institute of Technology, Haifa, Israel; ^2^Department of Sociology and Anthropology, Tel-Aviv University, Tel-Aviv, Israel; ^3^Curiosity Lab, Department of Industrial Engineering, Tel-Aviv University, Tel-Aviv, Israel; ^4^Sagol School of Neuroscience, Tel-Aviv University, Tel-Aviv, Israel

**Keywords:** social-robots, human-robot interaction, collaborative learning, active learning, educational technology, higher education

## Abstract

Acknowledging the benefits of active learning and the importance of collaboration skills, the higher education system has started to transform toward utilization of group activities into lecture hall culture. In this study, a novel interaction has been introduced, wherein a social robot facilitated a small collaborative group activity of students in higher education. Thirty-six students completed a 3 h activity that covered the main content of a course in Human Computer Interaction. In this within-subject study, the students worked in groups of four on three activities, moving between three conditions: instructor facilitation of several groups using pen and paper for the activity; tablets facilitation, also used for the activity; and robot facilitation, using tablets for the activity. The robot facilitated the activity by introducing the different tasks, ensuring proper time management, and encouraging discussion among the students. This study examined the effects of facilitation type on attitudes toward the activity facilitation, the group activity, and the robot, using quantitative, and qualitative measures. Overall students perceived the robot positively, as friendly and responsive, even though the robot did not directly respond to the students' verbal communications. While most survey items did not convey significant differences between the robot, tablet, or instructor, we found significant correlations between perceptions of the robot, and attitudes toward the activity facilitation, and the group activity. Qualitative data revealed the drawbacks and benefits of the robot, as well as its relative perceived advantages over a human facilitator, such as better time management, objectivity, and efficiency. These results suggest that the robot's complementary characteristics enable a higher quality learning environment, that corresponds with students' requirements and that a Robot Supportive Collaborative Learning (RSCL) is a promising novel paradigm for higher education.

## Introduction

Classrooms in the twenty-first century are slowly being transformed from frontal lectures halls filled with passive students, to collaborative small groups actively participating in project based learning (Helle et al., 2006; Kokotsaki et al., 2016). Studies have shown that such active participatory learning is more effective in content retention (Al-Balushi and Al-Aamri, 2014) and engagement (Fernandes et al., 2014). Thus, emphasis has been diverted to so-called twenty-first century skills (Crane, 2003; Saavedra and Opfer, 2012; Trilling and Fadel, 2012), with focus on the 4C super-skills, i.e., communication, collaboration, creativity, and critical thinking (Shulman, 1986; Kivunja, 2015). This focus has created several new pedagogies, such as to provide students with the opportunity, within the classroom, to observe, imitate, and practice *critical agency*, and reflect upon it (ten Dam and Volman, 2004); *collaborate* by learning to share tasks and resources and be responsible for their tasks (Lai et al., 2017); engage in inter-, trans-, and cross-disciplinary approaches to promote *creativity* (Harris and de Bruin, 2018); and use project-based learning as the basis for improving *communication* skills (Saenab et al., 2018).

In higher education, the proliferation of massive online open courses (MOOCs) (Bozkurt et al., 2017) has not lived up to its initial expectation (Khalil and Ebner, 2014; Thomas and Thorpe, 2019). However, the emergence of the “flipped classroom” paradigm (Gilboy et al., 2015; Schmidt and Ralph, 2016), in which students learn the material at home via on-line learning platforms and then discuss and practice it in small groups in the classroom, has been shown to be highly effective (Chen and Chen, 2015; Thomas and Thorpe, 2019).

These paradigms have started to reshape the role of the lecturer in higher education, wherein the role of group facilitator has become an important aspect of teaching in such scenarios (Franco and Nielsen, 2018). Group facilitation involves the mediation of the material via encouragement of communication, active participation, and discussion of all the group members (Phillips and Phillips, 1993). Best practices involve promotion of reflection and action (Franco and Nielsen, 2018) and maintaining engagement density (Matsuyama et al., 2015).

These changes to classic teaching methods have also introduced new challenges as large classrooms, restructured as several small discussion groups, demand the attention of the lecturer, and her TAs (Moust and Schmidt, 1994). While on-line discussion forums have prospered in recent years (Pendry and Salvatore, 2015; Yang et al., 2015; Chiu and Hew, 2018), with AI assisting in managing such forums (Goel and Joyner, 2017), studies have shown that personal face-to-face interactions and discussions in small groups have their advantages (Chen and Chen, 2015; Thomas and Thorpe, 2019). The question of scaling-up group facilitation is thus of prominent importance.

Concurrently, social robots have progressed drastically in the last decade, especially in the field of education (Mubin et al., 2013; Brown and Howard, 2014; Gordon et al., 2015; Belpaeme et al., 2018b). Compared to tablets and screens, social robots have been shown to convey more learning gains (Wainer et al., 2006; Leyzberg et al., 2012; Li, 2015; Luria et al., 2017) and evoke more emotional expressions (Spaulding et al., 2016). They have been used to teach science (Shiomi et al., 2015), math (Brown and Howard, 2014), languages (Kory and Breazeal, 2014; Belpaeme et al., 2015; Hein and Nathan-Roberts, 2018), and even nutrition (Short et al., 2014). Moreover, they have been used to promote meta-cognitive skills such as curiosity (Gordon et al., 2015; Ceha et al., 2019) and growth mindset (Park et al., 2017). Social robots in education have taken different roles. They have been used as peers or companions in learning with the students (Okita et al., 2009), or tutors in which the robot teaches students (Belpaeme et al., 2018b). Moreover, social robots have been used as teachers using frontal lecture mode (Sisman et al., 2018), one-on-one interaction (Short et al., 2014; Gordon et al., 2015) and even in two-person dialogues (Tahir et al., 2014). Several studies have addressed how a single robot can interact with small groups of children (Leite et al., 2015; Strohkorb et al., 2015), elderly (Matsuyama et al., 2008), and adults (Matsuyama et al., 2015). More specifically, several studies examined possible roles of social robots in group interaction (Jung et al., 2015; Shen et al., 2018; Alves-Oliveira et al., 2019; Correia et al., 2019; Oliveira et al., 2019).

These advances in social robots resulted in their slow introduction into the educational system (Belpaeme et al., 2018a; Kory-Westlund and Breazeal, 2019) and into homes (Scassellati et al., 2018). Many studies have focused on young children, from preschoolers (Kory and Breazeal, 2014), through elementary school (Leite et al., 2015), and adolescents (Björling et al., 2019), with special interest in children with Autism (Scassellati et al., 2018). In recent years, several applications of social robots in higher education have started to emerge (Brown and Howard, 2014; Edwards et al., 2016; Deublein et al., 2018). Pfeifer and Lugrin (2018) showed that a female robot can lead to better learning in female students while breaking stereotypical beliefs. Rosenberg-Kima et al. (2019) showed that social robots can serve as teaching assistants by answering simple questions of students working in small groups.

In this contribution we report on a higher education application of social robots as small group facilitators. Our goal was to compare the current state, in which an instructor attempts to facilitate several groups in the classroom, to a robot facilitator that is more limited in terms of emotional and cognitive capabilities yet remains with the group for the entire activity to facilitate it. An undergraduate course group activity that summarizes the material taught during a full semester has been converted into an interaction facilitated by a social robot, Nao, and mediated by tablets. Groups of four students performed the group activity, followed the instructions of the robot facilitator, discussed the material, and then answered questionnaires about the interaction. The same groups performed similar activities with tablets alone and with pen-and-paper, facilitated by the instructor of the course (within-subject design). Their impressions of the different activities' modalities are reported.

## Method

### Participants

Thirty-six students (age *M* = 28 years, SD = 0.3, 58.3% females) who participated in the course *Human Computer Interaction* completed a three-parts activity that covered the main content of the course and served as preparation for the final exam. The students consented to include their participation data in the study. The study was approved by the IRB.

### Materials

All the participants completed three group-linked activities, each covering different content of the course, and serving as training for the final exam. The students worked collaboratively in groups of four students (nine groups in total). The overall goal of the activities was to design a family App that aims to provide all the needed information and tools to support family communication and planning (e.g., weekly schedule, messages, budget planning, etc.), while enabling each member of the family to be an active participant. Each activity lasted about 30 min. The specific goals of each activity are described in Table 1.

**Table 1:** Description of the goals of each task in the Human-Computer Interaction course activities, where the overall goal was to develop a family-oriented App.

**Activity**	**Overall goals of each sub-task**
Activity 1: target audience	1.1. Defining the target audience of the application. The students were instructed to first work individually for 2 min and then combine the group members' lists into one list of two target audiences. 1.2. Building a survey: (a) given questions, identify different type of questions (e.g., questions appropriate for online survey, questions appropriate for focus groups, questions that might evoke confirmation bias etc). (b) Select five questions that fit the target audience of the application.
Activity 2: metaphors	2.1. Defining metaphors for the application. Again, the students were instructed to first work individually for 2 min and then combine the group members' lists into one list of two metaphors. 2.2. Screens: given a screenshot of an application, identify design features (e.g., centered, direct instruction, etc.)
Activity 3: interfaces	3.1. Defining interfaces for the application. Again, the students were instructed to first work individually for 2 min and then combine the group members' lists into one list of two interfaces. 3.2. Evaluating screens: rate two screens on a 1–5 rating scale on four given heuristics.

### Conditions

**Figure 1:**
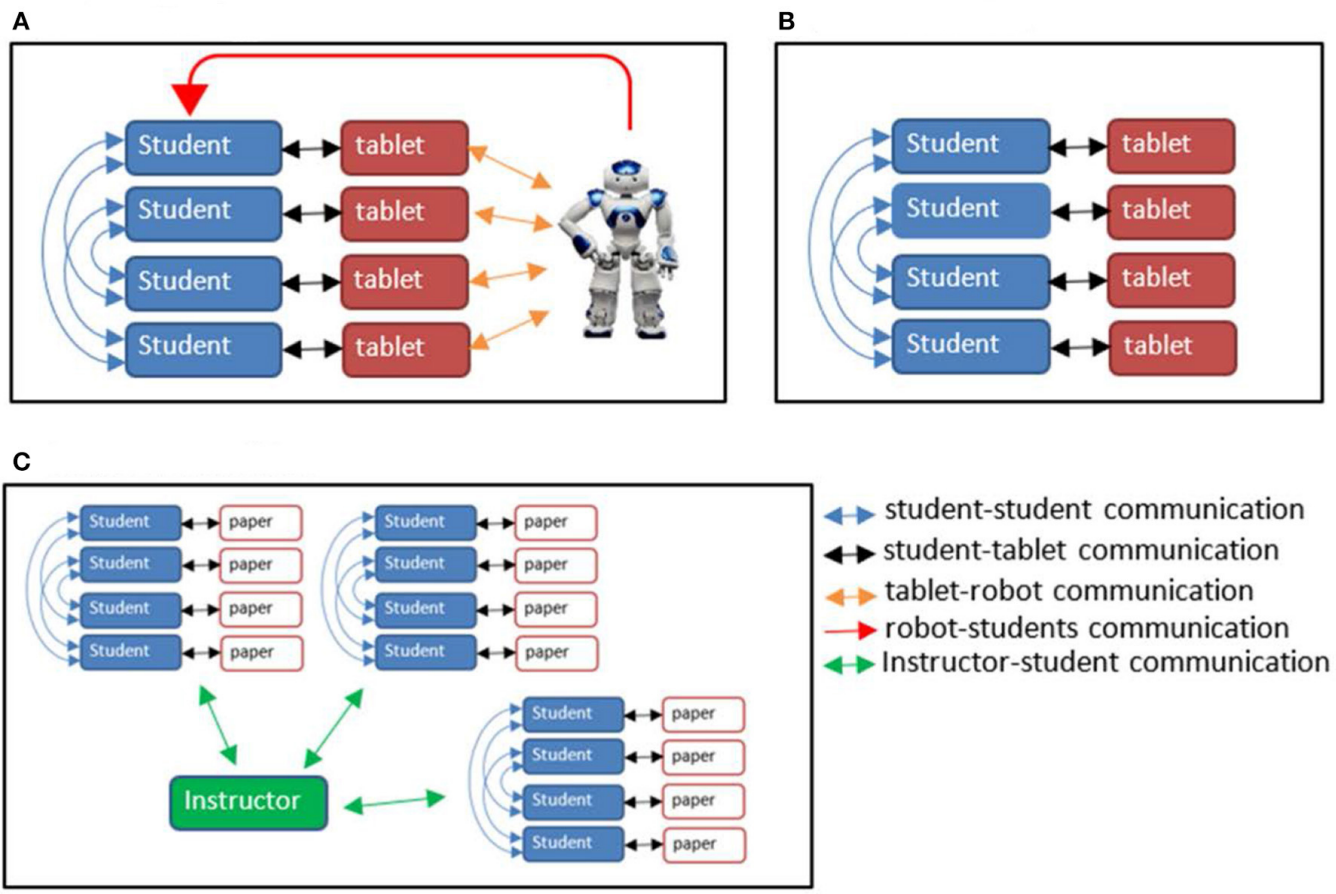
Setup architecture of the three conditions used in the study. **(A)** Robot condition. **(B)** Tablet condition. **(C)** Instructor condition.

The study had a within-subject design, wherein the students worked in groups of four, each group going through three conditions (Figure 1):

1. Robot condition: In this condition, each group of students performed the task using tablets and were facilitated by a social robot. Each group was located in a separate room.
2. Tablet condition: In this condition, the groups of students performed the task and were facilitated by tablets, located in a large lecture hall.
3. Instructor condition: In this condition, the groups of students performed the task using pen and paper. All the groups were located in a large lecture hall and were facilitated by a single instructor.

The order of the conditions differed between the groups, but the order of the activities with respect to the task itself was the same, as each activity was building on the previous one.

Each of the nine groups completed the three activities and experiences all three conditions. Thus, for example, the first group completed the first activity with a robot-facilitation (robot + tablets), then moved to a different room where it completed the second activity with a tablet-facilitation (tablets only), and lastly moved to a different room where it completed the third activity with paper based instruction, and an instructor-facilitation. The sequence of conditions varied between the groups to control for activity and order effect (see Table 2 for a complete sequence of all the groups).

**Table 2:** Sequence of activity and conditions for each group.

**Groups**	**Activity 1**	**Activity 2**	**Activity 3**
1–3	Robot	Tablets	Instructor
4–6	Instructor	Robot	Tablets
7–9	Tablets	Instructor	Robot

### Setup Architecture

The setup architecture of the social-robot facilitator (robotator) condition included communication between four students, four android tablets (one per student), and a NAO robot (see Figure 1A). Unfortunately, state-of-the-art Natural Language Processing (NLP) could not support verbal communication facilitation of a group at this level. Hence, the robot spoke to the students utilizing pre-recorded sentences, but in order to establish bidirectional communication, the tablets served as additional sources of input and output between the robot and students. To implement this architecture, we used Python and Kivy to develop the tablet application, and ROS (Robot Operating Systems) and Python to control and manage the communication between the Robot and the tablets. The robotator facilitated this interaction by introducing the different tasks, managing the time (e.g., the robot said in relation to the design App task: “take 2 min to list different target audiences for the App, and then create a combined list with two target audiences,” after which a timer of 2 min started followed by the next set of instructions), and encouraging discussion between the students (e.g., if two students answered the same question differently, the robot would say “I see that your answers are different, would you like to discuss that?”).

The setup architecture of the tablets-only condition included communication between four students, each with one tablet (see Figure 1B). Python and Kivy were used to develop the tablet application, that included presentation of the tasks, guidelines, and time management via a presented timer.

The setup for the instructor condition included exchange of ideas between four students who worked with paper-based instructions that included the exact same instructions as in the tablet and robot-tablet conditions, but did not include support such as a timer. A human instructor was present in the classroom to answer questions of all the groups in this condition.

### Measures

#### Attitude Toward the Robot\Tablet\Instructor Questionnaire

After each activity, students completed a 13-items questionnaire to evaluate their attitudes toward the robot\tablet\instructor depending on the condition. Students responded to a series of statements on a 5-point Likert-type scale (from 1 = Strongly disagree to 5 = Strongly agree) (e.g., “I trusted the information given by the robot\tablet\instructor”; see Table 3 for the complete list). The questionnaire items were combined to form the attitudes toward the facilitation scale (Cronbach's alpha = 0.881).

**Table 3:** Results for attitudes toward the activity facilitation questionnaire (A), attitudes toward the group activity questionnaire (B), and Godspeed questionnaires (C).

	**Statement**	**Robot**		**Tablet**		**Instructor**		p^*^	**η^2^**
		**M**	**SD**	**M**	**SD**	**M**	**SD**		
(A) Attitudes toward thefacilitation questionnaire	1. I understood the robot\app\Instructor	3.62	1.15	4.14	0.91	3.71	1.01	0.010	0.163
	2. The facilitation of the r\a\t was of high quality	3.47	1.08	3.59	0.94	3.42	0.85	0.083	0.221
	3. I trusted the information given by the robot\app\Instr.	3.71	1.06	4.18	0.87	3.93	0.87	0.023	0.145
	4. I felt comfortable with the robot\app\Instr. presence	4.09	0.83	3.97	0.72	3.78	0.81	0.586	0.040
	5. I felt comfortable with the behavior of the robot\app\Instr.	3.79	0.98	4.00	0.89	3.93	0.99	0.767	0.013
	6. The robot\app\Instr. adjusted to the class	3.62	0.89	3.69	0.96	3.40	1.13	0.204	0.062
	7. I would like more activities with the robot\app\Instr.	3.32	1.30	3.42	1.09	3.29	1.19	0.475	0.028
	8. The robot\app\Instr. responded to the group	2.94	1.18	3.11	1.32	3.47	1.15	0.168	0.069
	9. The robot\app\Instr. was friendly	3.79	1.01	3.69	0.87	3.84	1.04	0.750	0.011
	10. The robot\app\Instr. behaved human-like	2.71^a^	0.87	2.37^a^	0.81	3.84^b^	1.18	0.000^*^	0.544
	11. I liked the robot\app\Instr. facilitator	3.26	0.83	2.83	0.92	3.27	1.10	0.08	0.045
	12. The activity with the robot\app\Instr. was pleasant	3.91	0.83	3.49	0.85	3.65	0.91	0.177	0.064
	13. The activity with the robot\app\Instr. was interesting	3.94^a^	0.92	3.03^b^	0.98	3.23^b^	1.11	0.001^*^	0.226
	Attitudes toward the facilitation scale (items 1–13)	3.55	0.62	3.51	0.65	3.66	0.63	0.685	0.046
(B) Attitudes toward the group activity questionnaire	1. The group work contributed to understanding of the content	3.86	1.06	3.97	0.76	4.15	0.67	0.637	0.30
	2. I felt like I expressed myself during the discussions.	3.86	0.73	3.88	0.68	4.20	0.52	0.752	0.013
	3. All group members equally contributed to the discussion	3.74	1.01	3.53	0.96	3.90	1.02	0.636	0.030
	4. The work instructions were clear	3.00	1.21	3.53	1.08	3.65	0.99	0.519	0.043
	5. The contribution of the robot\tablet\Instr. was big	2.94	1.28	3.27	1.28	3.18	0.63	0.362	0.076
	6. I felt that the group members considered my opinions	4.17	0.75	4.12	0.48	4.35	0.62	0.597	0.034
	7. The sequence of tasks was logic and clear	3.60	1.09	3.91	0.93	3.85	0.93	0.712	0.022
	8. One group member managed most of the discussion	2.14	0.77	2.12	0.77	2.10	0.91	0.609	0.032
	9. I enjoyed working with my group members	4.29	0.57	4.03	0.83	4.40	0.60	0.554	0.039
	10. The group members felt free to express different opinions	4.37	0.73	4.32	0.73	4.50	0.60	0.944	0.004
	11. Group activities like this contribute to meaningful learning	3.77	0.94	3.65	0.84	3.89	0.74	0.552	0.042
	12. Group activities like this are a waste of time	2.26	0.98	2.50	0.75	2.25	0.85	0.895	0.007
	13. Group activities like this are superior to individual activities	3.57	0.95	3.62	0.74	3.70	1.08	0.523	0.042
	14. Groups activities contributes more than frontal lectures	3.51	0.82	3.67	0.96	3.70	0.92	0.139	0.123
	Attitude toward the group activity scale (items 1–7 and 9,10)	3.76	0.61	3.82	0.52	4.06	0.39	0.638	0.020
	General attitudes toward group activities scale (items 11–14)	3.65	0.69	3.62	0.56	3.75	0.64	0.599	0.036
(C) Godspeed questionnaires	Godspeed I: anthropomorphism	2.51	0.66	–	–	–	–	–	
	Godspeed II: animacy	2.66	0.65	–	–	–	–	–	
	Godspeed III: likable	3.64	0.73	–	–	–	–	–	
	Godspeed IV: perceived intelligence	3.15	0.72	–	–	–	–	–	
	Godspeed V: perceived safety	4.12	0.78	–	–	–	–	–	

* *Bonferroni adjusted alpha value of 0.002 (0.05/14) was used for the single items*.

*Bold value of p indicates a significant difference (given the Bonferroni correction) between ^a^ and ^b^*.

#### Attitude Toward the Group Questionnaire

After each activity, students completed a 14-items questionnaire to evaluate their attitudes toward the group. Students responded to a series of statements on a 5-point Likert-type scale (from 1 = Strongly disagree to 5 = Strongly agree). The questionnaire resulted in two subscales: attitudes toward the specific group activity scale (e.g., “The group work contributed to understanding the content”; see Table 3 for the complete list), which included items 1–10 excluding item 8 (Cronbach's alpha = 0.816), and attitudes toward group activities scale (e.g., “Group activities like this, are superior to individual activities”), which included items 11–14 (Cronbach's alpha = 0.743).

#### Godspeed Questionnaires

After the robot-facilitated activity, the students completed the 24-items Godspeed questionnaire, in which students responded to pairs of words and rated the robot on a 5-point semantic differential scale (e.g., Unfriendly-Friendly, Ignorant-Knowledgeable), resulting in 5 subscales: (I) Anthropomorphism consisting of 5 items (in this study Cronbach's alpha = 0.686), (II) Animacy consisting of six items (in this study Cronbach's alpha = 0.728), (III) Likable consisting of five items (in this study Cronbach's alpha = 0.867), (IV) Perceived Intelligence consisting of five items (in this study Cronbach's alpha = 0.845), and (V) Perceived Emotional safety (e.g., anxious vs. relaxed) consisting of three items (in this study Cronbach's alpha = 0.786) (Bartneck et al., 2009).

#### Qualitative Data

A semi open-ended questionnaire was used to collect qualitative data. The participants were asked to specify, in writing, three advantages, and three disadvantages the robot had as a facilitator of student groups. The open-ended questionnaire served as a means to get the perspective of students in their own words to provide “depth, detail, and meaning at a very personal level of experience” (Patton, 2014, p. 24). Nevertheless, given the limitations of an open-ended questionnaire in writing (e.g., dependent on writing skills of respondents or the impossibility of extending responses), observational data, based on video recording of activity, and a video sample analysis was used as a supportive tool to capture the context (Bauer and Gaskell, 2000).

### Procedure

**Figure 2:**
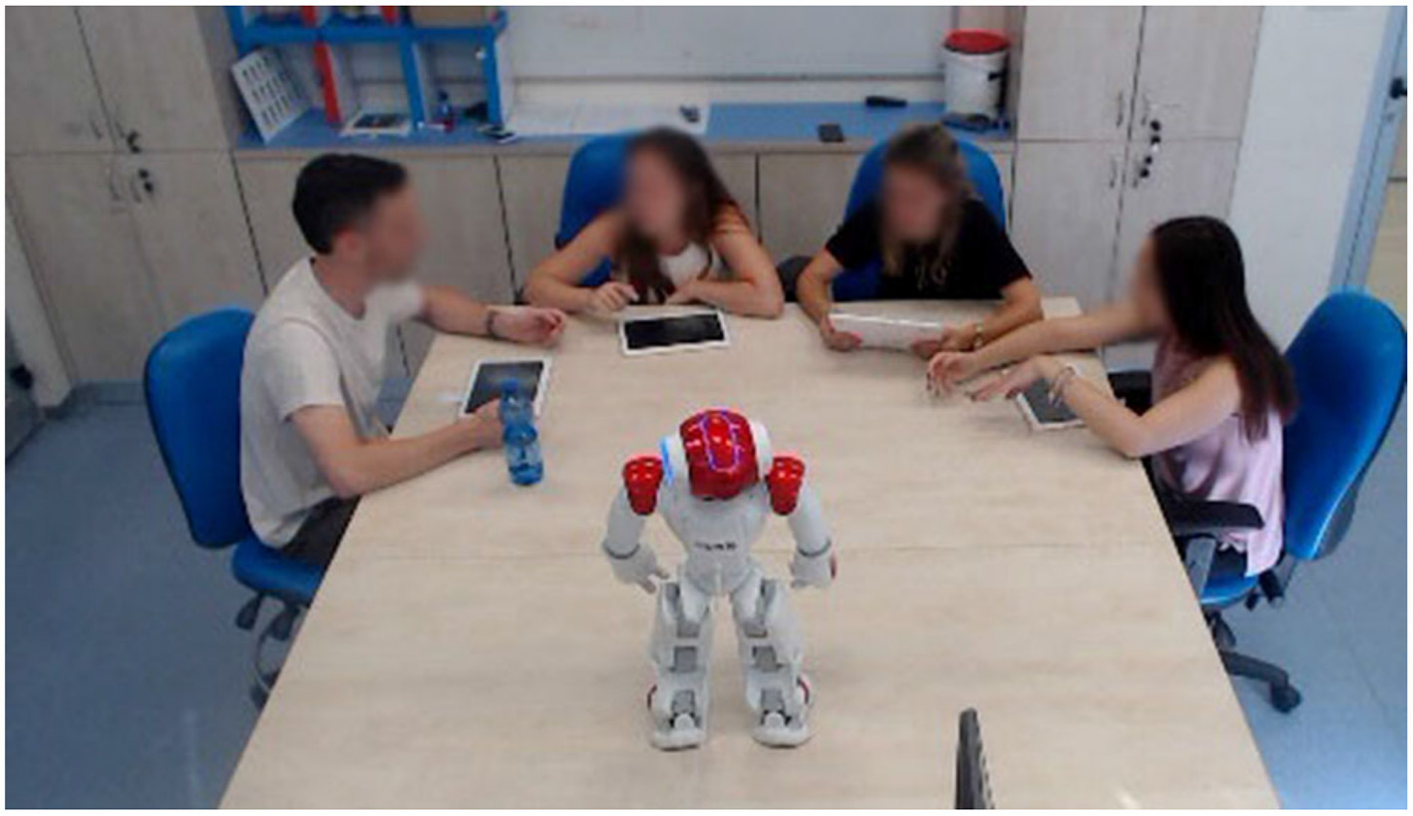
Footage from the study of the robot condition settings.

After signing a consent form, the students were placed in groups of four students. The groups were then guided to the location of their first activity settings according to their conditions as described in Table 2. Thus, groups 1–3 were guided to three different rooms in which the robots and tablets setting was located, groups 4–6 were placed in groups in one big room, where each student received a tablet, and groups 7–9 were placed in groups in one big room, where each student received paper-based instructions and a human instructor was present to answer questions. After completing the first activity, which took about 30 min, the students completed the questionnaires for about 15 min and were then guided to the location of the second activity according to the conditions, completed the second activity, filled again the questionnaires and were guided in the same way to the third activity. Overall completing the three activities, filling the questionnaires after each activity, and changing locations took 3 h (see Figure 2 for footage of the robot condition).

## Results

This study examined the effects of facilitation type (robot facilitation, tablet facilitation, and instructor facilitation) on attitudes toward the activity and attitudes toward the group activity using one-way within-subject ANCOVA with group order as a covariant to control for order and activity (in groups 1–3 the robot facilitated the first activity, in groups 4–6 the robot facilitated the second activity, and in groups 7–9 the robot facilitated the third activity). Overall, we did not find an effect for the group order. In addition, attitudes toward the robot were measured using the Godspeed questionnaires and were correlated to the attitudes toward the robot facilitation and the attitudes toward the robot group activity.

Preliminary data analysis included examination of missing data and outliers, verification of the equivalence of treatment groups, and tests for assumptions of the parametric statistics. Some of the students missed some of the items in which case they were omitted from the analysis in the relevant places. Shapiro–Wilk normality test was used to detect violation of the normal distribution assumption. Results indicated that several dependent measures were not normally distributed. Nevertheless, it was suggested that ANOVA is robust enough to moderate violations of this assumption (Blanca et al., 2017). The overall scales were normally distributed. In addition, Bonferroni correction was applied to adjust the alpha values: Bonferroni adjusted alpha value of 0.002 (0.05/14) was used for the single items.

### Attitudes Toward the Activity Facilitation

**Figure 3:**
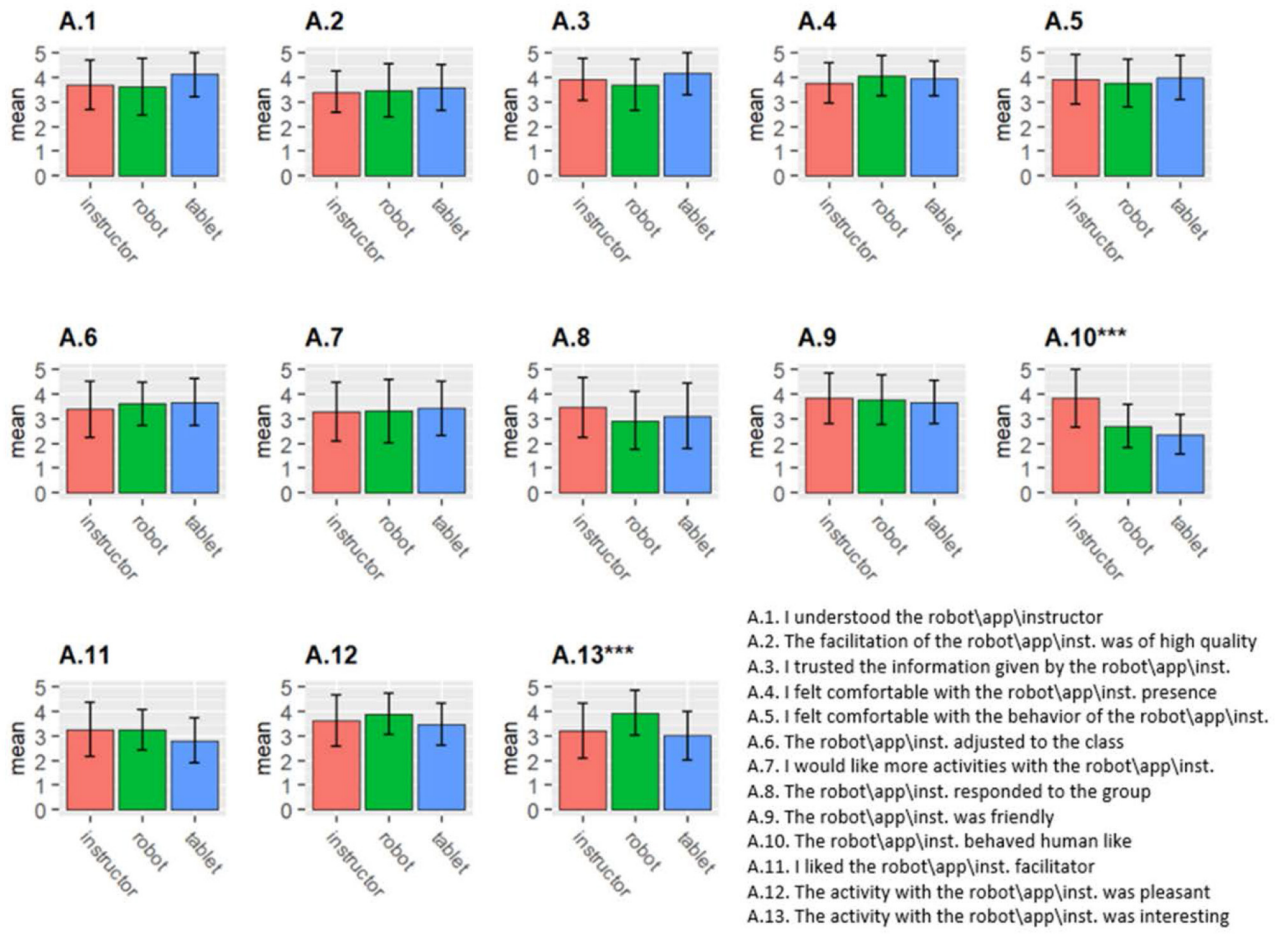
Attitudes toward the facilitation (see Table 3 for the complete list of statements). ***p < 0.001.

Overall the students reported that the activity with the robot was pleasant and interesting and the overall mean for the attitudes toward the robot facilitation scale was 3.55 (±0.62) (see Table 3A and Figure 3). Results of the within-subject ANCOVA for item 10 (“the robot\tablet\instructor behaved human-like”) indicated a significant within-subject effect [*F*_(2,52)_ = 30.982, *p* < 0.001]. *Post-hoc* Bonferroni tests revealed a significant difference between the instructor and the robot conditions (*p* < 0.001) and between the instructor and the tablet condition (*p* < 0.001). As expected, the participants rated the instructor as significantly more human-like than the robot and the tablet. There was no significant difference between the robot and the tablet condition. In addition, results of the ANCOVA for item 13 (“the activity with the robot\tablet\instructor was interesting”) indicated a significant within-subject effect [*F*_(2,52)_ = 7.576, *p* = 0.001]. *Post-hoc* Bonferroni tests revealed a significant difference between the robot and the tablet conditions (*p* = 0.004) and between the robot and the instructor condition (*p* = 0.023). The participants rated the activity with the robot as significantly more interesting than the tablet and the instructor conditions. There was no significant difference between the instructor and the tablet condition. Nevertheless, for the rest of the items there was no significance difference between the robot, the tablet, and the human instructor.

### Attitudes Toward the Collaborative Group Activity

Overall students rated the group activity positively (see Table 3B). Results of the within-subject ANCOVA yielded no significance effects. Attitudes toward the robot facilitator had an overall mean of 3.76 (±0.61) for the attitudes toward the current group activity scale and an overall mean of 3.65 (±0.69) for the attitudes toward general group activities scale.

### Attitudes Toward the Robot

**Figure 4:**
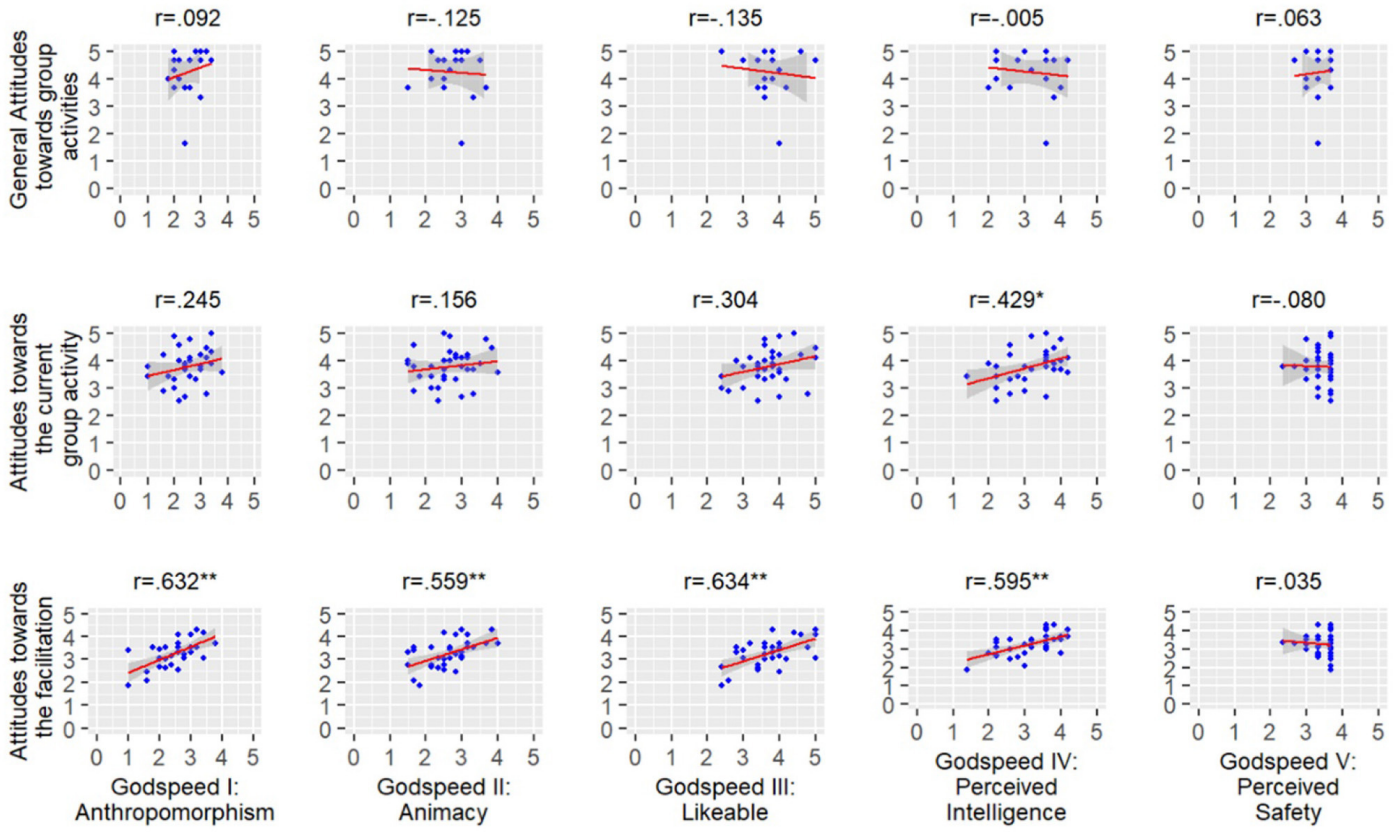
Pearson correlations between Godspeed questionnaires and the attitudes scales. **p < 0.01, *p < 0.05.

Godspeed questionnaire, consisting of five subscales, was used to measure the participants' attitudes toward the robot used in the study. On a 1–5 scale, overall the participants rated the robot 2.51 (±0.66) on anthropomorphism, 2.66 (±0.65) on animacy, 3.64 (±0.73) on likable, 3.15 (±0.72) on perceived intelligence, and 4.12 (±0.78) on perceived safety (see Table 3C). We were interested in finding what were the correlations between the Godspeed subscales and the three attitudes scales (attitudes toward the facilitation scale, the group activity scale, and group activities scale). With regard to the attitudes toward the facilitation scale, Pearson correlation tests indicated strong correlations between the scale and Anthropomorphism (*r* = 0.632, *p* < 0.01), Animacy (*r* = 0.559, *p* < 0.01), Likable (*r* = 0.634, *p* < 0.01), and Perceived Intelligence (*r* = 0.595, *p* < 0.01), but not with Perceived Safety (*r* = −0.080, *p* = 0.655). With regard to the attitudes toward the current group activity scale, only Perceived Intelligence of the robot was significantly correlated to the scale (*r* = 0.429, *p* < 0.05). With regard to attitudes toward general groups activities scale, none of Godspeed subscales was correlated to this scale (See Figure 4).

### Qualitative Results

Thematic analysis method (Boyatzis, 1998) was used for analyzing and reporting themes within the data. The method is applicable to the research objective to report the ways individuals make meaning of their experience, on the one hand, and is not wedded to any pre-existing theoretical framework, on the other (Braun and Clarke, 2006). Following the template approach (Crabtree and Miller, 1998), and based on a preliminary scanning of the data, 157 students' statements were classified by two research team members to four principle categories: (1) Technical Functionality Benefits. (2) Social and Psychological Benefits. (3) Technical Functionality Drawbacks. (4) Social and Psychological Drawbacks. Within each category, statements were re-reviewed, collating statements into relevant themes. The analysis according to the aforementioned coding resulted in a total of 48% statements expressing benefits the robot had as a facilitator of student groups vs. 52% statements expressing drawbacks. Results analysis to the semi-open question indicated that students were well-attentive to the interaction with the robot (supported by video recording analysis), to its benefits as well as to the drawbacks of using a robot as a group facilitator. Excluding apparent novelty effect statements revealed that they were more concerned with technical functionalities issues, but also attentive to social, and psychological aspects.

Themes that emerged in the category *technical functionality benefits* include efficiency (e.g., “saves manpower,” “time efficient,” “put the activity to order”)^1^, focus (e.g., “its mind is not distracted,” “focused on the tasks,” “concentrated only in issues relevant to the task”), accurate (e.g., “accurate instructions,” “don't forget anything”), and responsive (e.g., “responsive to topics addressed by the students via tablets”). Themes that emerged in the category *technical functionality drawbacks* include limited communication skills (e.g., “its voice was not clear enough,” “did not respond to oral questions,” “one-shot answer, cannot repeat it”), limited pedagogical skills (e.g., “you cannot ask it follow-on questions,” “instructions were not always clear”), and technical problems (“there were some bugs,” “its voice was not load enough,” “slow boot”).

Themes that emerged in the category *psychological drawbacks* include being inhuman (e.g., “not human,” “mechanic,” “frigid”), awkward (e.g., “strange eye contact,” “caused strange feelings,” “strange head movements”), limited communication skills (“did not interact enough with the group,” “did not adjust itself to the group,” “behaved in a not socially acceptable manner”), and impersonal (“no personal relationship”). Interestingly, for many students the fact that the robot was not human was an advantage. Thus, themes that emerged in the category *social and psychological benefits* include objective, not judgmental (e.g., “the robot have no personal bias against one of the students,” “the robot does not have a favorite student”), friendly (e.g., “the robot was cute and friendly”), pleasant (e.g., “the robot was polite,” “the robot was nice”). The themes *break routine* and *innovative* also emerged but were removed as they were related to the novelty effect.

In addition, analysis of the video recordings revealed that the robot served as a focal point and was very effective in facilitating the activity in terms of time management and group interaction. For example, when the robot gave the students 2 min for individual thinking, the students worked individually, and when it asked to regroup the students immediately regrouped and started to work together. In the tablet and instructor conditions, there was less of a clear distinction between individual and group activity. Thus, for example, when the students read the instruction to work individually for 2 min, in many cases they did not do that but rather worked in a group or pairs.

## Discussion

A novel interaction has been introduced, wherein a robot facilitated a small group activity of students in higher education. While we have not explicitly implemented a “flipped-classroom” paradigm (Gilboy et al., 2015; Thomas and Thorpe, 2019), since the students learned the material in a frontal lecture mode, we have applied principles of group facilitation to robot-directed interaction (Chen and Chen, 2015).

The post-interaction questionnaire and its quantitative analysis revealed interesting insights into the interaction. Most items did not reveal significant differences between instructor and robot. The only highly significant differences were the expected questions of perception of the robot/tablet/ instructor as human-like, and the perception of the activity as interesting. In the human-like perception question, students rated the instructor as obviously more human-like, but the difference between tablet and robot, while not small, was not significant. This may represent the perception of the students that the robot was “a machine,” much like a tablet, and not strictly “a social agent,” like a human (Kahn et al., 2011). The perception of the activity as “interesting” was rated significantly higher for the robot condition, but this may be due to the novelty effect: this was the first interaction of the students with a social robot.

Moreover, even though the robot did not directly respond to the students' verbal communications, they still perceived it as friendly and responsive. However, these results should be taken in view of the similar ratings the tablet-condition received. It is unsure how students interpreted “the app was friendly,” whereas “the robot was friendly” had a much more direct social interpretation.

The Godspeed questionnaire produced several important insights. The student's perception of the robots correlated with how they perceived the activities. However, the strongest correlations were between the perceived intelligence, anthropomorphism, animacy, likeability, and the facilitation itself. Hence, students who perceived the robot as more animate and likable, rated the facilitation higher. This conforms to previous studies with human facilitators that stressed the importance of the social presence of the facilitator on the activity (Franco and Nielsen, 2018). The rating of the current group activity was only correlated to the perceived intelligence of the robot, emphasizing the difference between activity, which relates to intelligence, and facilitation of the group, which relates also to animacy and likeability. In contrast, the perception of the robot was uncorrelated to the students' attitudes toward group activities in general. The robot's safety, while rated very high, did not correlate to any other scale. This may be due to the physical distance of the robot from the students, its more childlike appearance or lack of possibly threatening actions.

The qualitative analysis of the students' answers gave insights into the benefits of the current setup and raised issues that can be addressed in future applications. First, there were many benefits to the setup, e.g., time management which is an important concern in effective group activities (Gresalfi et al., 2012), accuracy and focus, which can add another layer of efficiency to repeated activities. Second, the fact that the robot was non-judgmental, as opposed to a human facilitator, raises the interesting topic of the benefits of social robots over humans in roles that involve possible judgments (Kidd and Breazeal, 2007). These results also support the media-equation according to which people relate to computers and other technologies, and in this case to robots, in the same way they relate to other human beings (Reeves and Nass, 1996).

However, many drawbacks shed light on possible improvements for future applications. The most obvious ones are technical, e.g., improved quality assurance tests on a larger scale setup are required. The biggest drawback that the students' commented on was the lack of communication skills and responsiveness. Due to technological challenges of natural language processing in a group scenario, especially in the students' native language, these lacks in the setup will not be overcome easily in the near future. However, improved perceptions, such as speaker recognition and engagement via facial expressions (Bhattacharya et al., 2018) can be implemented in such a setup and supply better social and emotional management for the group activity (Matsuyama et al., 2015). Overall, the students commented on the potential of this setup in terms of saving manpower and scalability, non-judgmental and objective facilitation, and increased focus and efficiency of activity management.

Considering the relative acceptance of the students of a robot facilitator puts the role of the future instructor in a new light (Franco and Nielsen, 2018). In our envisioned future “robot facilitated flipped classroom” paradigm, the group facilitation will be conducted by social robots. However, due to formidable technological challenges, the robot cannot understand the discussion's verbal content, nor deal with delicate emotional and social scenarios. Hence, the role of the robot could include for example time management and role assignment whereas the role of future instructors may focus more on answering complex questions, managing divergence from proper discussion content and dealing with emotional and social aspects of the task.

## Limitations

Several limitations in this study should be noted. First, the interaction with a social robot facilitator was novel for all the students and a novelty effect was evident especially with respect to some benefits noted by the students. In order to get a deeper understanding of the long-lasting potential of a social robot facilitator longer interventions (e.g., lasting over a semester) should be examined. In addition, this study was holistic. We were interested in comparing the current state of an instructor facilitating several groups in parallel to the scenario where several robots assist the instructor in facilitating the groups. Nevertheless, this holistic comparison comes with a price tag of control. Thus, there were several differences between the conditions: students in the paper-based condition sat in a lecture hall with all the other groups, whereas in the robot condition they were alone with the robot in a separate room. This makes it more difficult to claim that the effect was of a robot vs. human, or the fact that it was a private facilitator (the robot) vs. a shared facilitator (the instructor). Yet another limitation was the lack of pre-post exams of the content that was due to the fact the HCI content involves skills that are hard to measure. Future studies should conduct a research in a content area that is easier to assess for learning.

## Conclusions

We have introduced a novel educational paradigm in which a social robot facilitated a small group activity in higher educational settings. We have conducted a first study that compared the robot-facilitated setup to human facilitation and activities with tablets only. We have shown that while human facilitation is still considered better in most aspects, students could tap into the benefits of a robot facilitator, such as better time management, objectivity, and efficiency. Nevertheless, in terms of the quantitative data we did not find significant differences that cannot be attributed to a novelty effect (e.g., the robot was significantly more interesting).

Future work will include upgrading the setup to include augmented perception via a larger sensor suite composed of directional microphones and cameras. This will enable real-time speaker recognition and engagement detection to facilitate also the social and emotional sides of the group activity. Furthermore, applying the setup in primary and secondary educational settings raises new challenges, and new opportunities.

Furthermore, while the current study did not asses the students' communication and collaboration skills, future studies will examine the possible positive influence of repeated robot facilitation, using state-of-the-art pedagogy, such as time-management, and maintaining engagement density (Matsuyama et al., 2015), on students' 4C's super skills (Shulman, 1986; Kivunja, 2015).

To conclude, this contribution offers a new and exciting venue for using social robots for robot supported collaborative learning (RSCL) in education, as efficient, objective, and social facilitators for small group discussions.

## Data Availability Statement

The datasets generated for this study are available on request to the corresponding author.

## Ethics Statement

The studies involving human participants were reviewed and approved by Tel Aviv University Internal Review Board. The patients/participants provided their written informed consent to participate in this study.

## Author Contributions

RR-K contributed to the code of the setup, ran the study, and contributed to the data analyzes and writing of the paper. YK assisted in the execution of the research and the analysis of the qualitative findings. GG contributed to the code of the setup, helped in running the study, and contributed to the writing of the paper.

### Conflict of Interest

The authors declare that the research was conducted in the absence of any commercial or financial relationships that could be construed as a potential conflict of interest.

## Footnotes

^1^Translated from Hebrew by team members.
